# Test-retest reliability of physical activity questionnaires in Parkinson’s disease

**DOI:** 10.1186/s12883-021-02426-y

**Published:** 2021-10-15

**Authors:** Samuel Ånfors, Ann-Sofi Kammerlind, Maria H. Nilsson

**Affiliations:** 1 Department of Rehabilitation Medicine, Jönköping, Region Jönköping County, and Department of Health, Medicine and Caring Sciences, Linköping University, Linköping, Sweden; 2 Department of Health Sciences, Lund University, Lund, Sweden; 3 Futurum, Region Jönköping County, and Department of Health, Medicine and Caring Sciences, Linköping University, Linköping, Sweden; 4 Memory Clinic, Skåne University Hospital, Malmö, Sweden; 5 Clinical Memory Research Unit, Department of Clinical Sciences Malmö, Lund University, Lund, Sweden

**Keywords:** Parkinson disease, Exercise, Reproducibility of results, Surveys and questionnaires

## Abstract

**Background:**

People with Parkinson’s disease are less physically active than controls. It is important to promote physical activity, which can be assessed using different methods. Subjective measures include physical activity questionnaires, which are easy and cheap to administer in clinical practice. Knowledge of the psychometric properties of physical activity questionnaires for people with Parkinson’s disease is limited. The aim of this study was to evaluate the test-retest reliability of physical activity questionnaires in individuals with Parkinson’s disease without cognitive impairment.

**Methods:**

Forty-nine individuals with Parkinson’s disease without cognitive impairment participated in a test-retest reliability study. At two outpatient visits 8 days apart, the participants completed comprehensive questionnaires and single-item questions: International Physical Activity Questionnaire-Short Form (IPAQ-SF), Physical Activity Scale for the Elderly (PASE), Saltin-Grimby Physical Activity Level Scale (SGPALS) and Health on Equal Terms (HOET). Test-retest reliability was evaluated using the intraclass correlation coefficient (ICC), standard error of measurement (SEM), limits of agreement, weighted kappa or the Svensson method.

**Results:**

Several of the physical activity questionnaires had relatively low test-retest reliability, including the comprehensive questionnaires (IPAQ-SF and PASE). Total physical activity according to IPAQ-SF had an ICC value of 0.46 (95% confidence interval [CI], 0.21–0.66) and SEM was 2891 MET-min/week. The PASE total score had an ICC value of 0.66 (95% CI, 0.46–0.79), whereas the SEM was 30 points. The single-item scales of SGPALS-past six months (SGPALS-6 m) and HOET question 1 (HOET-q1) with longer time frames (6 or 12 months, respectively) showed better results. Weighted kappa values were 0.64 (95% CI, 0.45–0.83) for SGPALS-6 m and 0.60 (95% CI, 0.39–0.80) for HOET-q1, whereas the single-item questions with a shorter recall period had kappa values < 0.40.

**Conclusions:**

Single-item questions with a longer time frame (6 or 12 months) for physical activity were shown to be more reliable than multi-item questionnaires such as the IPAQ-SF and PASE in individuals with Parkinson’s disease without cognitive impairments. There is a need to develop a core outcome set to measure physical activity in people with Parkinson’s disease, and there might be a need to develop new physical activity questionnaires.

## Background

People with Parkinson’s disease (PD) are less physically active and spend more time in sedentary activities than controls [1, 2], even at the early stages of the disease [2]. In comparison with controls, fewer people with PD [2, 3] reach the World Health Organization’s global health recommendations of 150 min of moderate- to vigorous-intensity physical activity (PA) per week [4]. Disease severity, walking ability, and disability in daily living explain most of the decreased PA in PD, but falls, fear of falling, comorbidity, and depression are also associated with less PA [1]. Low outcome expectation, lack of time, fear of falling, non-motor symptoms, and lack of support are examples of barriers to exercise [5]. According to the European guidelines for physiotherapists who treat people with PD, a key goal is to prevent inactivity and promote PA [6].

PA is defined as “any bodily movement produced by skeletal muscles that results in energy expenditure” [7]. Exercise is PA that is planned, structured, and aims to improve or maintain physical fitness [7]. There is a dose-response relationship between the level of PA and the risk for developing PD [8]. Whether PA alters the disease prognosis remains to be shown, but the health benefits are undoubted [9]. For example, exercise improves muscle strength, balance, and motor symptoms in people with PD [10, 11]. It is important to investigate the level of PA in order to prevent physical inactivity and evaluate the effect of PA interventions.

PA can be assessed using criteria, objective and subjective measures. Criteria measures include measuring heat production, oxygen consumption, or production of carbon dioxide. Such measures are not feasible in clinical practice. Examples of objective measures include accelerometers, pedometers, and heart frequency measurements. Unlike pedometers, accelerometers can measure the intensity of PA. Accelerometers are commonly used in research. Nevertheless, they have limitations in measuring PA without acceleration (e.g., bicycling), activities with higher energy consumption (e.g., climbing stairs), and when PA is performed in water such as swimming [12]. Examples of subjective measures are PA questionnaires (PAQs) and PA diaries. PAQs are suitable for use in larger epidemiologic studies and are commonly used in clinical practice because they are easier and cheaper to administer than objective measures [12, 13]. For a comprehensive assessment of total PA, all domains of PA should be included in the questionnaire such as sports and household activities [14]. Knowledge of the psychometric properties of PAQs for people with PD is limited. For example, no specific PAQ is recommended in the European physiotherapy guidelines for PD [6]. Reliable and valid measures are required for use in clinical practice and research. Moreover, the psychometric properties of an instrument are sample dependent [15].

## Methods

The aim of this study was to evaluate the test-retest reliability of PAQs in people with PD without cognitive impairment. The evaluation included both comprehensive questionnaires and single-item questions: International Physical Activity Questionnaire-Short Form (IPAQ-SF), Physical Activity Scale for the Elderly (PASE), Saltin-Grimby Physical Activity Level Scale (SGPALS), and Health on Equal Terms (HOET).

### Participants

All patients diagnosed with idiopathic PD living in Region Jönköping County, Sweden, who received care at the Internal Medicine or Geriatric departments at County Hospital Ryhov, Jönköping, Sweden, were considered eligible for inclusion. Exclusion criteria were other neurologic disorders, dementia diagnosis, documented cognitive impairments (e.g. mild cognitive disorder or subject to cognitive investigation), using a wheelchair indoors, or insufficient understanding of the Swedish language. The sole use of patient-reported outcome measures among cognitively impaired respondents may introduce additional challenges [16], therefore those who scored < 26 points on the Montreal Cognitive Assessment (MoCA) [17] at the first study visit were also excluded. MoCA is a valid instrument for detecting mild cognitive impairment and dementia in individuals with PD [18]. Participants with other co-morbidities were not excluded.

### Recruitment procedure

**Fig. 1:**
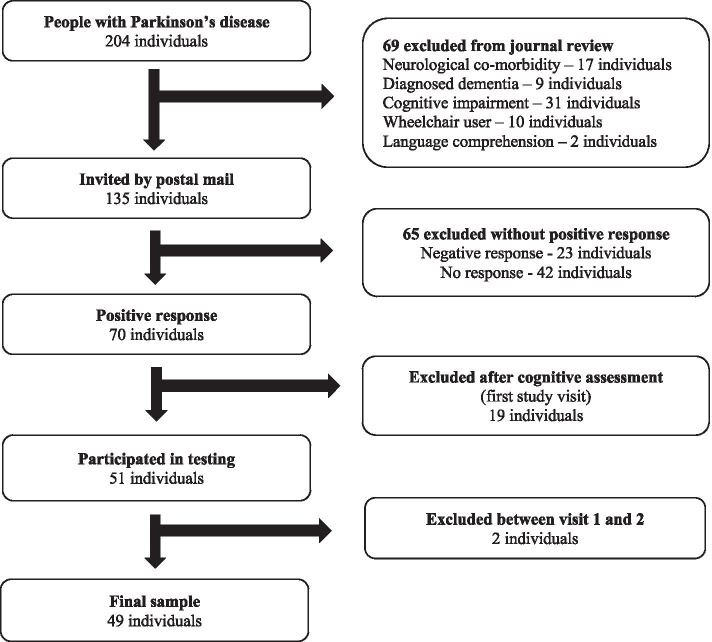
Flowchart of the recruitment procedure

Potential participants were identified using an existing database for patients diagnosed with PD in Region Jönköping County and by screening medical records. The recruitment procedure is presented in Fig. 1. In total, 204 individuals diagnosed with PD were identified, and 69 (mean age, 71 years; standard deviation [SD], 10.1 years; 67% men) were excluded according to the study criteria (Fig. 1). The remaining 135 people with PD received information about the study, a letter of inquiry, and a pre-stamped return envelope by post.

Ninety-three people responded (response rate, 69%). The mean age of those who did not respond (*n* = 42) or declined to participate (*n* = 23) was 68 years (SD, 12.3 years) and a median duration of PD of 4.4 years (q1–q3, 1.4–8.0 years); 51% were men. The mean age of the 70 participants (63% men) who accepted the invitation was 67 years (SD, 7.7 years) and the median duration of PD was 4.3 years (q1–q3, 1.7–8.8 years). There were no significant differences (*P* ≥ 0.15) in relation to sex, age, or duration of PD between those who did not respond or declined versus those who accepted the invitation.

Those who agreed to participate were contacted by phone; they received further information about the study and were invited to an outpatient visit that included cognitive screening. An additional 19 people (78% men) were excluded because their MoCA score was < 26; their median age was 71 years (q1–q3, 65–77 years) and their median duration of PD was 3.3 years (q1–q3, 1.4–9.6 years). After the initial visit, one participant withdrew from the study and another sustained a fracture and could not participate. This yielded a final sample of 49 participants: 27 men (55%) and 22 women (45%). The mean age was 65 years (SD, 6.9 years) and a median duration of PD 4.3 years (q1–q3, 1.9–7.1 years). The participants’ characteristics are presented in Table 1.

**Table 1:** Characteristics and descriptive information for the sample (*N* = 49)

Sex; men/women, n (%)	27 (55)/22 (45)
Age (years), mean (SD)	65 (7)
PD duration (years), median (q1–q3)	4.3 (1.9–7.1)
PD severity (H&Y), median (q1–q3)	2 (2–2)
Motor symptoms (UPDRS III), median (q1–q3)^a^	14 (7–19)
Cognitive impairments (MoCA), median (q1–q3)	28 (27–29)
Body mass index (kg/m^2^), mean (SD)	26.8 (4.6)
Physical capacity (6-min walk test; meters), mean (SD)	451 (109)
Comorbidity, self-reported (yes), n (%)	22 (45) ^b^
Heart and vascular disease, n (%)	10 (20)
Goiter/hypothyroidism, n (%)	3 (6)
Osteoarthritis, n (%)	4 (8)
Pulmonary disease, n (%)	3 (6)
Disease in gastrointestinal or bladder function, n (%)	3 (6)
Diabetes, n (%)	1 (2)
Inguinal hernia, n (%)	1 (2)
Using walking aid indoors, n (%)	5 (10)
Using walking aid outdoors, n (%)	12 (24)
Freezing of gait (scoring ≥1 on item 3 of the FOGQsa), n (%)	17 (35)
Falls in the past 6 months, n (%)	10 (20)
Physical activity, accelerometry data^c^	
Sedentary time (min/day), median (q1–q3)^d^	598 (534–670)
Light PA (min/day), median (q1–q3)^e^	166 (112–214)
Lifestyle PA (min/day), median (q1–q3)^f^	31 (21–51)
Moderate PA (min/day), median (q1–q3)^g^	10 (2–20)
Vigorous PA (min/day), median (q1–q3)^h^	0 (0–0)
Very vigorous PA (min/day), median (q1–q3)^i^	0 (0–0)

Reported % is valid %

*SD* standard deviation, *PD* Parkinson’s disease, *q1–q3* first and third quartiles, *H&Y* Hoehn and Yahr staging scale (score range, 1–5; higher = worse), *UPDRS III* Unified PD Rating Scale, part III – motor examination (score range, 0–108; higher = worse), *MoCA* the Montreal Cognitive Assessment (score range, 0–30; higher = better), *FOGQsa* self-administered version of the Freezing of Gait questionnaire, *PA* physical activity

^a^4 missing values

^b^3 of the 22 participants had > 1 comorbidity

^c^n = 46 with valid data

^d^ 0–99 cpm

^e^100–759 cpm

^f^ 760–1951 cpm

^g^1952–5724 cpm

^h^5725–9498 cpm

^i^ > 9498 cpm

### Data collection procedure

The participants were booked for two outpatient visits 8 days apart (median, 8.0 days; min-max, 8–10 days). All participants were examined by the same assessor (SÅ). At both visits, the following PAQs were self-administered in the following order: two versions of SGPALS [19, 20]; two questions from HOET [21, 22]; IPAQ-SF [23], and PASE [24]. SGPALS was administered in two versions using two different time frames for retrospective recall: SGPALS 1 week (SGPALS-w) and SGPALS 6 months (SGPALS-6 m).

At the first visit, the participants were given a booklet of self-administered questions for descriptive purposes. In addition, clinical assessments addressed global cognitive function, motor symptoms, disease severity, and physical capacity. For descriptive purposes and between the two visits, the participants wore an accelerometer: ActiGraph GT1M (ActiGraph LLC, Pensacola, FL, USA) [25], which provides uni-axial measurements of daily PA. The participants wore the accelerometer (placed on the right hip) during waking hours for 7 days, except for activities in water. The accelerometer data are presented in Table 1.

At the second visit, the participants responded to all four PAQs. In order to investigate whether the participants were stable at retest [26], they were given questions on whether they had undergone any changes in medication or deep brain stimulation, answered with yes/no. They also answered questions about changes since their first visit in relation to their walking ability, physical capacity, and/or PA. These questions had five response options: “much better/higher”, “better/higher”, “unchanged”, “worse/lower”, or “much worse/lower”.

### Physical activity questionnaires

#### IPAQ-SF

IPAQ-SF is a self-administered questionnaire, which was created to enable international comparisons of PA [23]. It includes 7 items that cover daily PAs with regard to transportation, work, and household and leisure time. The questions refer to the past 7 days. The following data were registered in relation to vigorous and moderate activities as well as for walking and sedentary time: the number of days that these activities took place and the mean time per day. A sum of metabolic equivalent task (MET) minutes/week was calculated and the respondents were categorized into groups of low, moderate, or high PA [27]. MET reflects energy expenditure in different PAs [28], and one MET equals the energy consumption at rest. Activities with higher intensity are given higher MET values and when multiplied by frequency and duration, they are presented as MET-minutes per day or per week [29].

IPAQ-SF is one of the most widely used questionnaires on PA, and several studies have evaluated its psychometric properties although not in people with PD [27]. Test-retest reliability of the total PA time (IPAQ-SF) was reported in a study using a pooled Spearman coefficient (0.76). The sample consisted of 1974 generally middle-aged people from 12 countries [23]. Another study of 49 residents in Hong Kong, aged 15–55 years, reported an intraclass correlation coefficient (ICC) of 0.79 [30]. The items on PA level showed inconsistent test-retest reliability results with the lowest ICC value of 0.30 for moderate PA in a group of 108 Norwegian men aged 20–39 years [31]. ICC values for vigorous PA have ranged from 0.61 [31] to 0.75 [30]. ICC values have ranged from 0.80 [31] to 0.97 [30] for sedentary time.

#### PASE

PASE includes 12 items (ordinal) that cover PA during the past 7 days in three different domains: (1) leisure, (2) household, and (3) work-related activities. It was developed for an elderly population (≥65 years) and includes questions on PA with lower energy consumption (e.g., gardening and walking). The different activities are weighted for estimated energy expenditure, with higher weights for more vigorous activities. It yields subscores for the three domains, which are summarized to a total PASE score (range, 0–360 or above). Although PASE includes sedentary activities, these are not included in the total score. Test-retest reliability studies of PASE total score among older people without cognitive impairments reported ICC values ranging from 0.65 to 0.81 [32–34]. ICC values for its subscales have also been reported for older people, although cognitive status was not mentioned: leisure time PA (0.56), household PA (0.94), and work-related PA (0.91) [35]. So far, the psychometric properties of PASE have not been evaluated in people with PD.

#### SGPALS

SGPALS is a single-item questionnaire; it aims to identify individuals with a sedentary lifestyle and higher risk profile [36]. It originally covered four levels of PA in relation to sport and leisure [20], but has since been modified to a six-grade scale that also includes domestic activities [19]. The six response categories range from “hardly any physical activity” to “hard or very hard exercise regularly and several times a week, where the physical exertion is great, such as jogging or skiing” [37]. The modified scale was used in this study. Two versions were administered in order to cover different time frames of retrospective recall. The initial question was then revised regarding the time frame: “How much did you move and exert yourself physically during the past week (SGPALS-w)/six months (SGPALS-6 m)?” Test-retest reliability of the six-grade version of SGPALS has been studied in a Finnish project among elderly people, with Pearson correlation coefficients between 0.62 and 0.66 [38, 39].

#### HOET

Since 2004, the Public Health Agency in Sweden has conducted annual surveys called “Health on Equal Terms” [21]. Two questions concern PA. The first question (HOET-q1) is phrased as follows: “How many times have you exercised and exerted yourself physically in your free time during the past 12 months?” It addresses the leisure time domain and has four response categories (i.e., ordinal scale): “sedentary free time”, “moderate exercise in free time”, “moderate regular exercise in free time”, and “regular exercise and training”. The second question (HOET-q2) is phrased “How many hours in a normal week do you do moderately strenuous activities that make you warm?” This is one of the questions of IPAQ-SF, which has been changed to an ordinal scale with five response alternatives and it concerns “a normal week” instead of the past 7 days. The response alternatives are in reverse order compared with HOET-q1: “5 hours a week or more”, “more than 3 hours but less than 5 hours a week”, “between 1 and 3 hours a week”, “at most 1 hour a week”, and “not at all”. HOET-q2 aims to categorize sufficiently active persons (> 30 min or > 60 min of daily PA on a moderate level) from persons with a sedentary lifestyle [22]. Neither HOET-q1 nor HOET-q2 seems to have been evaluated in relation to test-retest reliability (i.e., regardless of the sample).

### Additional descriptive data

Descriptive data included age, sex, and duration of PD (years). Self-administered questions concerned years of education, comorbidity, use of walking aids (indoors and outdoors), a history of falls during the past 6 months and freezing of gait (FOG). FOG was assessed according to item 3 (score 0–4, higher = worse) of the self-administered version [40] of the FOG questionnaire [41] (i.e. FOGQsa). Those scoring ≥1 were categorized as “freezers” [42]. The participant’s weight was measured with a weighing scale (Philips HF 351/00) [43], whereas height was self-reported.

Several clinical assessments were included. Motor symptoms were assessed according to part III of the Unified Parkinson’s Disease Rating Scale (UPDRS) [44]; the total score ranges from 0 to 108 points (higher scores = worse). The severity of PD was assessed according to the Hoehn and Yahr staging scale, which ranges from I to V (higher = worse) [45, 46]. Global cognitive functioning was assessed according to MoCA; the maximal score is 30 points and results ≥26 points are considered normal [17]. Physical capacity was tested with the Six-Minute Walk Test [47]; the participants walked for 6 min (fast speed) and the total distance (meters) was measured.

### Analysis

Normally distributed data are presented as means and standard deviation, whereas non-normally distributed data are presented as medians and first and third quartiles.

Regarding accelerometry, at least 10 h of wear time a day and 4 valid days were required to get valid data for analysis. This follows recommendations for use in research settings [48, 49]. ActiLife software (ActiGraph LLC, Pensacola, FL, USA) was used to process the data [50]. Data were collected in epochs of 10 s and summarized to 60 s for analysis of the data. There is no consensus on which algorithms to use when analyzing. In accordance with Choi et al. [51], 90 min of no activity was considered nonwear time, allowing a spike tolerance of 2 min with non-zero counts.

Test-retest reliability analyses were done using different methods, depending on the nature of the data and to gain a comprehensive analysis. The ICC, two-way mixed, absolute agreement, [52] was used in relation to the continuous scales of IPAQ-SF and PASE. ICC values were calculated for their respective subscales as well as for the total score. For group-level comparisons, acceptable ICC values should exceed 0.70 [16, 53]. If used for decisions on an individual level, it has been suggested that the ICC value should be at least 0.90 [16]. In addition, to obtain an absolute value of the measurement error, the standard error of measurement (SEM) was calculated using the formula: $\mathrm{SEM}=\mathrm{SDtest}\ 1\times \sqrt{1-\mathrm{ICC}}.$

Moreover, limits of agreement (LoA) were visualized with Bland-Altman plots [54] for the differences and means of the paired data in PASE and IPAQ-SF, a method recommended when evaluating questionnaires [55]. In this study, the purpose was to mark the width of the measurement error and detect proportional bias.

Quadratic weighted kappa was used for the ordinal data of SGPALS and HOET. Landis and Koch [56] have proposed the following as standards for strength of agreement for the kappa coefficient: 0.01 = poor, 0.01–0.20 = slight, 0.21–0.40 = fair, 0.41–0.60 = moderate, 0.61–0.80 = substantial, and 0.81–1.00 = almost perfect.

The statistical methods of ICC and kappa are recommended in reliability studies of patient-reported outcome measures [53]. They were complemented by a method described by Svensson [57] (Örebro University, Sweden) for testing the stability of paired ordinal data, which provides a more comprehensive analysis. According to the Svensson method, systematic disagreement and additional individual variations are measured and expressed in terms of percentage agreement (PAgr), relative position (RP), relative concentration (RC), and relative rank variance (RV) [57]. PAgr explains the proportion of accurately matched paired answers with no change over time, values ranging from 0 to 100; a value of 100 indicates full agreement and high values are preferable. RP reflects whether a systematic disagreement between test and retest is present on a group level or not. RC measures any shift in concentration from test to retest, that is, if the paired answers are clustered differently. For RP and RC, the results range from − 1 to 1; the desirable value is close to 0. The 95% confidence interval (CI) should include 0 to be considered a stable value for the paired data. For RV, the results can range between 0 and 1, with preferable values close to 0 indicating a homogeneous individual change. High RV values indicate greater individual variance, independent of the systematic disagreement on a group level [57]. The Svensson method was used for the ordinal data of SGPALS and HOET. *P* < 0.05 was considered a significant difference.

SPSS software, version 20 (SPSS Inc., Chicago, IL, USA.) [58] was used for the statistical analysis. Weighted kappa was analyzed with SAS (SAS Institute Inc., Cary, NC, USA) [59] and Svensson’s method with a program provided on an internet web page [60].

## Results

No statistically significant differences in PA scores were found between test and retest in any of the PAQs. Six participants (12%) had missing data on SGPALS-6 m; there were no missing data for the remaining PAQs. Moreover, 45 of 49 (92%) participants reported that their walking ability as well as physical capacity was unchanged between test and retest. PA was reported as changed by 11 participants (22%); 2 much higher, 5 higher, and 4 lower. They had no significant differences between test and retest in any of the PAQs examined. Sensitivity analysis was done with these 11 participants excluded, and the results were comparable with no decisive differences in the reliability analysis. Therefore, all 49 participants were included in the study.

### Physical activity level

**Fig. 2:**
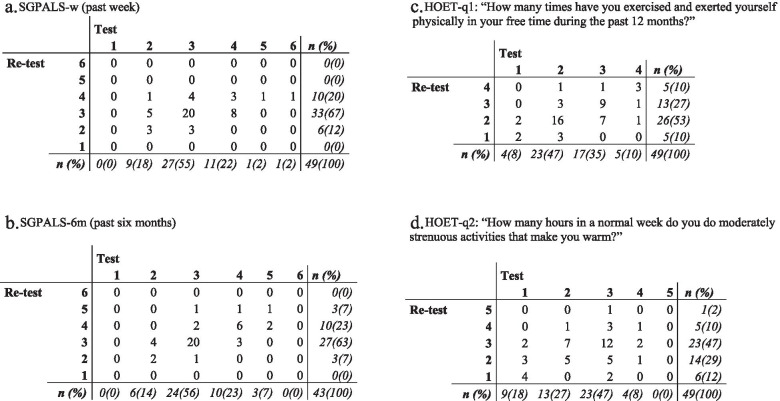
Crosstabs for ordinal scales. Legend: This figure presents crosstabs for responses at test and retest for SGPALS and HOET, i.e. in relation to each of their response categories. SGPALS has six response categories (higher values = more vigorous physical activity). HOET-q1 has four response categories (higher values = more vigorous physical activity). HOET-q2 has five response categories (higher values = less vigorous physical activity). HOET-q1, Health on Equal Terms question 1; HOET-q2, Health on Equal Terms question 2; SGPALS-6 m, Saltin-Grimby Physical Activity Level Scale past six months; SGPALS-w, Saltin-Grimby Physical Activity Level Scale past week

The participants were physically active mostly at a moderate level, according to the assessment with IPAQ-SF and PASE (Table 2). The IPAQ-SF results (test and retest) resulted in categorization of the respondents into low (31 and 24%), moderate (43 and 47%), and high (27 and 29%) PA. Most (63 and 69%) had no vigorous PA at all. According to PASE, they got most of their PA from household-related activities: 68 and 73% in the test and retest, respectively. The ordinal scales of SGPALS and HOET resulted in a concentration of participants categorized in a moderate level of PA (Fig. 2). SGPALS categorized none at level 1 (“hardly any PA”) and very few at level 5–6 (vigorous PA). The results of HOET were better spread but still with nearly half of the participants at a moderate level of PA. The accelerometer data showed even less PA with a dominance of sedentary time, indicating a discrepancy between the PAQs and the accelerometers (Table 1). Valid accelerometer data were obtained from 46 participants (94%).

**Table 2:** Test-retest reliability of IPAQ-SF and PASE in people with Parkinson’s disease (N = 49)

	Test 1		Test 2		ICC (95% CI)	SEM^a^
	Median (q1–q3)	Mean (SD)	Median (q1–q3)	Mean (SD)		
IPAQ-SF						
Vigorous, MET-min/week	0 (0–480)	559 (1268)	0 (0–600)	642 (1309)	0.37 (0.09–0.59)	1010
Moderate, MET-min/week	480 (0–1440)	1593 (2958)	480 (0–1260)	1132 (1663)	0.36 (0.10–0.58)	2360
Walking, MET-min/week	594 (264–1040)	777 (905)	594 (297–1040)	928 (1087)	0.51 (0.27–0.69)	634
Sedentary time, min/week	1890 (1260–2100)	1933 (960)	1680 (1260–2100)	1852 (925)	0.60 (0.38–0.75)	610
Total, MET-min/week	1173 (744–3592)	2930 (3950)	1674 (804–3822)	2702 (2726)	0.46 (0.21–0.66)	2891
PASE						
Leisure time subscore	19 (7–33)	23 (19)	15 (8–28)	24 (27)	0.49 (0.25–0.68)	14
Household subscore	85 (50–106)	78 (40)	85 (50–91)	79 (34)	0.69 (0.51–0.81)	22
Work subscore	0 (0–0)	16 (40)	0 (0–0)	10 (25)	0.30 (0.02–0.53)	33
Total score	118 (81–153)	117 (51)	104 (75–142)	113 (53)	0.66 (0.46–0.79)	30

IPAQ-SF, International Physical Activity Questionnaire, short form; PASE, Physical Activity Scale for the Elderly; q1–q3, first and third quartiles; SD, standard deviation; ICC, two-way mixed intraclass correlation coefficient; CI, confidence interval; SEM, standard error of measurement; MET, metabolic equivalent task

^a^SEM defined as $\mathrm{SEM}=\mathrm{SDtest}\ 1\times \sqrt{1-\mathrm{ICC}}$

### Test-retest reliability of IPAQ-SF and PASE

The ICC values for the subscales of IPAQ-SF ranged from 0.36 for moderate PA to 0.60 for sedentary time. Total PA had an ICC value of 0.46 and SEM was 2891 MET-min/week (Table 2). Thus, in nearly all aspects, IPAQ-SF presented low test-retest reliability with high measurement error.

ICC values for the subscales of PASE ranged from 0.30 for work-related PA to 0.69 for household-related PA. The PASE total score had an ICC value of 0.66, whereas the SEM score was 30 points (Table 2).

**Fig. 3:**
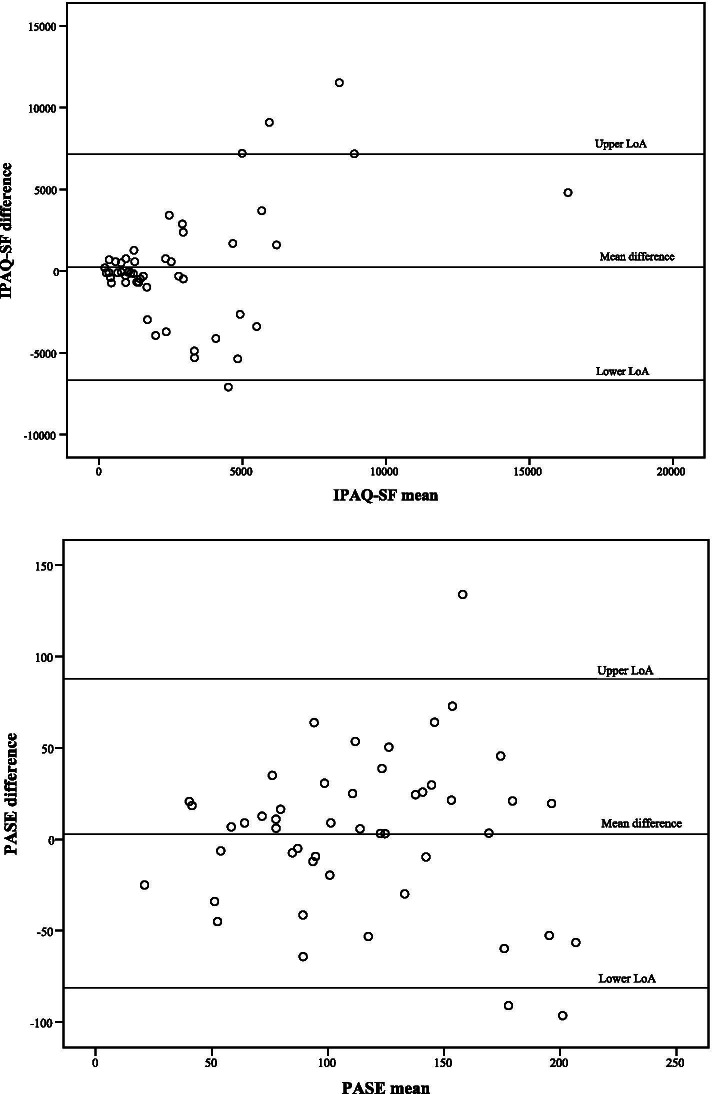
Limits of agreement with Bland-Altman plots for the total scores for IPAQ-SF and PASE. Legend: Each dot visualizes the difference in two measurements (test and retest; y-axis) from the same individual, and in relation to the average value of the two measurements (x-axis) for this individual. The mid horizontal line is drawn at the mean difference, which is the estimated bias. The lines above and below represent the upper and lower limits of agreement (defined as the mean difference +/− 1.96 x SD of the differences). The results for IPAQ-SF are presented as the sum of MET-minutes/week (higher values = more vigorous physical activity). The total PASE score can range from 0 to 360 (or above, higher values = more vigorous physical activity). IPAQ-SF, International Physical Activity Questionnaire, short form; LoA, limit of agreement; PASE, Physical Activity Scale for the Elderly; SD, standard deviation

LoAs are presented with Bland-Altman plots for the differences and means of the paired data in the total scores of IPAQ-SF and PASE (Fig. 3). Both total scales and their subscales show heteroscedastic values (i.e. the magnitude of differences increases proportionally to the size of the measurement), except for household PA in PASE. For the other comparisons, the difference between test and retest values increased with higher mean values. The mean difference (SD) for total PA in IPAQ-SF was 228 (3526) and LoA − 6684 and 7140 MET-min/week. PASE total score had a mean difference (SD) score of 3 (43) and LoA − 81 and 88. For total PA in IPAQ-SF, 5 outliers (10%) exceeded the LoA, whereas PASE total score had 3 outliers (6%) (Fig. 3).

### Test-retest reliability of SGPALS and HOET

The test-retest results of SGPALS and HOET are presented in Table 3. According to weighted kappa values, SGPALS-6 m (0.64) and HOET-q1 (0.60) had substantial and moderate agreement, respectively. SGPALS-w and HOET-q2 had weighted kappa values < 0.40. The Svensson method showed best PAgr for SGPALS-6 m (67%) followed by HOET-q1 (61%) (Table 3). The RP and RC were close to zero for both versions of SGPALS as well as for both HOET questions, indicating no systematic disagreement on a group level. Moreover, RV results had a CI that included zero except for HOET-q2 (0.16; 95% CI, 0.04–0.29); this is a result of individual variability. The best reliability results were found for SGPALS-6 m and HOET-q1.

**Table 3:** Test-retest reliability of SGPALS and HOET (N = 49)

		**Test 1: median (q1–q3)**	**Test 2: median (q1–q3)**	**wKappa (95% CI)**
SGPALS-w^a^		3 (3–4)	3 (3–3)	0.39 (0.18–0.61)
SGPALS-6 m^a,b^		3 (3–4)	3 (3–4)	0.64 (0.45–0.83)
HOET-q1^c^		2 (2–3)	2 (2–3)	0.60 (0.39–0.80)
HOET-q2^d^		3 (2–3)	3 (2–3)	0.39 (0.16–0.62)
	**PAgr (%)**	**RP (95% CI)**	**RC (95% CI)**	**RV (95% CI)**
SGPALS-w	53	−0.01 (−0.17 to 0.14)	0.13 (− 0.01 to 0.26)	0.08 (0.00–0.19)
SGPALS-6 m	67	0.05 (−0.07 to 0.17)	0.08 (−0.07 to 0.22)	0.02 (0.00–0.04)
HOET-q1	61	−0.08 (− 0.21 to 0.05)	− 0.03 (− 0.17 to 0.12)	0.04 (0.00–0.10)
HOET-q2	45	0.08 (−0.08 to 0.25)	0.01 (−0.17 to 0.19)	0.16 (0.04–0.29)

SGPALS, Saltin-Grimby Physical Activity Level Scale; HOET, Health on Equal Terms; wKappa, kappa with quadratic weighting; q1–q3, first and third quartiles; CI, confidence interval; SGPALS-w, SGPALS past week; SGPALS-6 m, SGPALS past six months; HOET-q1, HOET question 1; HOET-q2, HOET question 2; PAgr, percentage agreement; RP, relative position; RC, relative concentration; RV, relative rank variance; PA, physical activity

^a^Score 1–6, higher = more PA

^b^n = 43 (6 missing)

^c^Score 1–4, higher = more PA

^d^Score 1–5, higher = less PA

## Discussion

This is the first evaluation of test-retest reliability in people with PD for the PAQs used in this study.

The main results of this study are that several of the PAQs had relatively low test-retest reliability, including the comprehensive questionnaires of IPAQ-SF and PASE. Best results were found for the single-item scales, SGPALS-6 m and HOET-q1, which use longer time frames (6 or 12 months).

### Test-retest reliability results

IPAQ-SF did not reach acceptable test-retest reliability with lower ICC values than recommended [16, 53], and the SEM value almost exceeded the mean value. Previous studies of a general population aged 15–65 years reported acceptable reliability for total PA (coefficients of 0.76–0.79 [23, 30]), but one of these studies reported Spearman correlations [23]. This discrepancy in findings probably reflects sample differences. The present findings may not be so surprising because IPAQ-SF was developed for younger respondents (18–65 years) [23].

PASE did not reach the limit of acceptable test-retest reliability, although the total PASE score had an ICC value of 0.66, which is close to the recommended cutoff of 0.70 [16, 53]. Because PASE was developed for use with older people ≥65 years of age [24], we had anticipated better results. Previous studies of healthy older persons reported ICC values ranging from 0.79 to 0.99 for PASE total score, and PASE has been recommended for use in groups of older adults [14]. Although test-retest reliability has been acceptable in different disease-specific samples [61–63], this is the first study to evaluate this in a PD sample. Moreover, the SEM value was 30 points and the LoAs were wide. These findings corroborate previous studies that reported a large measurement error in relation to PASE [34, 61–63]. However, in nearly all aspects, PASE showed better reliability results than IPAQ-SF.

Only a few previous studies of SGPALS and no studies of HOET have evaluated test-retest reliability. Of the PAQs used in this study, only SGPALS-6 m and HOET-q1 had acceptable results for test-retest reliability. Both are single-item questions. This is a surprising finding because scales with multiple items have been suggested to render better reliability results [16]. The present findings are difficult to explain. It might be that single-item questions are easier to comprehend and/or that one should preferably use longer periods for retrospective recall in relation to PA for this population. The latter is further supported by the fact that SGPALS-w and HOET-q2, which both have a 1-week time frame, showed low kappa results.

When choosing PAQs, the purpose must be clear; that is, whether the aim is to categorize individuals according to PA level or whether the aim is to evaluate changes over time or the effect of an intervention. The ordinal scales of SGPALS-6 m and HOET-q1 seem to be suitable for categorizing individuals into groups of different activity levels for research purposes or in clinical settings. Although single item questions are easy to use, it needs to be noted that we lose detailed information as compared to multi-item instruments, and they are less sensitive for detecting changes in types of activity and intensity (e.g. over time or intervention effects) [64]. Our findings suggest that it is preferable to complement PAQs with objective measures of PA, such as using accelerometers, for example in intervention studies.

### Physical activity level and future perspectives

Our data showed low levels of PA in people with PD, consistent with previous studies [1–3]. However, one study showed that PASE scores did not differ significantly from controls when only including participants with an early PD (i.e. mean PD duration was 16.6 months) [65]. This underlines the importance of focusing on maintaining PA after diagnosis.

The participants in our study were relatively young with mild PD without cognitive impairments, and they still had a sedentary lifestyle. This is important knowledge because promoting PA is one of the key components for physical therapists who treat individuals with PD [6]. For assessment of PA in people with PD, it is important to choose measures that include low energy PA, because the participants were physically active mostly at a moderate level. Moreover, they got most of their PA from household-related activities, which underlines the importance of including such activities when evaluating PA. We recommend developing a core outcome set [66] for measuring PA in people with PD, with a consensus on what concepts of PA to assess and how to assess it. There might even be a need to develop new subjective measures.

### Methodological considerations: strengths and limitations

This study addresses the paucity of psychometric studies in relation to PAQs within the field of PD, and the knowledge gained is anticipated to be important for both clinicians and researchers. A strength is that test-retest reliability was comprehensively investigated in relation to several PAQs. Moreover, the final sample size of 49 participants is close to the recommendation of 50 participants for reliability studies [67, 68]. However, we do acknowledge that other psychometric properties are also important.

Participants with cognitive impairments were excluded, which affects the external validity of the present findings. That is, our findings are not valid for people with PD with cognitive impairments, and mild cognitive impairment is common in those with PD [69]. The reasoning for excluding people with cognitive impairments was that subjective measures and patient-reported outcomes require good cognitive functioning for reliable results due to retrospective recall [70]. Therefore, as an initial step, we focused on evaluating test-retest reliability of PAQs in individuals with PD without cognitive impairments.

Eight days were used between test and retest, which implies that two different weeks were the objects of the PAQs. This might give biased results because PA varies from day to day, between weeks, and during seasons of the year [71–73]. However, we detected no statistically significant difference in PA between test and retest. A shorter period between test and retest has been associated with a higher reliability coefficient [74]. If the time span is too long, a greater risk for an actual change occurs. At retest and as recommended [26], specific questions addressed whether the participant had undergone or perceived any changes since the first administration of the PAQs. Eleven participants reported a change in PA between test and retest (no statistical differences in PAQ scores): 7 reported a higher PA level and 4 a lower PA level. The fact that more participants reported a higher PA level at retest might mirror that they had been using accelerometers, possible motivators to increase their PA. This should be kept in mind in future studies.

Participants with PD can have both motor and non-motor fluctuations. Although we did include specific questions that addressed whether the participant had undergone or perceived any changes since the first administration of the PAQs, it would have been of value to include descriptive data of both motor and non-motor symptoms also at retest.

## Conclusions

Single-item questions with a longer time frame (6 or 12 months) for PA were shown to be more reliable than multi-item questionnaires such as the IPAQ-SF and PASE in people with PD without cognitive impairments. There is a need to develop a core outcome set for measuring PA in people with PD, and there might be a need to develop new PAQs.

## Data Availability

All relevant data are within the manuscript. The dataset generated and analyzed in the current study is not publicly available due to privacy constraints relating to the ethical approval and informed consent signed by the participants. Data sharing was not stated in the informed consent signed by the participants.

## Abbreviations

CI: Confidence interval
FOG: Freezing of gait
FOGQsa: the self-administered version of the FOG questionnaire
HOET: Health on Equal Terms
HOET-q1: HOET question 1
HOET-q2: HOET question 2
ICC: Intraclass correlation coefficient
IPAQ-SF: International Physical Activity Questionnaire-Short Form
LoA: Limit of agreement
MET: Metabolic equivalent task
MoCa: Montreal Cognitive Assessment
PA: Physical activity
PAgr: Percentage agreement
PAQ: PA questionnaire
PASE: Physical Activity Scale for the Elderly
PD: Parkinson’s disease
RC: Relative concentration
RP: Relative position
RV: Relative rank variance
SD: Standard deviation
SEM: Standard error of measurement
SGPALS: Saltin-Grimby Physical Activity Level Scale
SGPALS-6 m: SGPALS past six months
SGPALS-w: SGPALS past week
UPDRS: Unified Parkinson’s Disease Rating Scale
